# Lymphocele Containing Staphylococcus lugdunensis

**DOI:** 10.7759/cureus.11666

**Published:** 2020-11-23

**Authors:** Dinesh Keerty, Manoj Das, Timothy N Hembree, Asha Ramsakal, Elizabeth Haynes

**Affiliations:** 1 Internal and Hospital Medicine, Moffitt Cancer Center, Tampa, USA; 2 Internal Medicine/Nephrology, Geisinger Health System, Danville, USA; 3 Internal Medicine, Moffitt Cancer Center, Tampa, USA

**Keywords:** coagulase-negative staphylococcus, lymphocele, bacteremia, staphylococcus lugdunesis

## Abstract

Staphylococcus species are a leading cause of community-acquired bacteremia. Of them, the most serious cause of mortality is from methicillin-resistant Staphylococcus aureus, with mortality rates as high as 40%. Another Staphylococcus species that has been noted to cause equal levels of infection and mortality is Staphylococcus lugdunensis (S. lugdunensis). It can cause harmless skin infections to life-threatening endocardial complications. We would like to report a rare presentation of S. lugdunensis bacteremia from a lymphocele that developed post surgery. An 80-year-old male presented to the emergency department with complaints of abdominal pain and fevers. Cultures of lymphocele fluid grew S. lugdunensis. A computed tomography of the abdomen and pelvis with contrast showed the presence of a large lymphocele. S. lugdunensis is a coagulase-negative staphylococci normally known to be a skin colonizer. Over the years, it has shown to cause a wide variety of infections especially involving prosthetic joints and heart valves. S. lugdunensis has been noted to be highly susceptible to penicillins, such as oxacillin, erythromycin, linezolid and a wide a variety of other antibiotics. S. lugdunensis produces a biofilm that makes treatment challenging even with susceptible antibiotics. However, the data on S. lugdunensis is growing as more case reports are being published in regards to source and susceptibilities.

## Introduction

Staphylococcus species are a leading cause of community-acquired bacteremia. Of them, the most serious cause of mortality is from methicillin-resistant Staphylococcus aureus, with mortality rates as high as 40% [1]. Another Staphylococcus species that been noted to cause equal levels of infection and mortality is Staphylococcus lugdunensis (S. lugdunensis). It can cause harmless skin infections to life-threatening endocardial complications [2]. We would like to report a rare presentation of S. lugdunensis bacteremia from a lymphocele that developed post surgery.

## Case presentation

An 80-year-old male presented to the emergency department with complaints of abdominal pain and fevers. The patient’s past medical history is significant for bladder cancer. One month ago, he underwent a radical cystoprostatectomy with ileal conduit placement. Prior to surgery, he was treated with neoadjuvant nivolumab and ipililumab. He stated that over the past two weeks, he had been having abdominal pain and fevers. The fevers were gradual in onset, intermittent, with a max temperature of less than 100.4°F. Along with the fevers, he complained of left flank and groin pain, aching, which worsened from positional changes. He was taking Tylenol for the pain and fevers for the past two weeks. He denied any signs of hematuria, dysuria, nausea, vomiting, or diarrhea. He denied any recent travels or sick contacts. The patient reported adequate output from the ileal conduit. The patient denied any nausea, vomiting, diarrhea, constipation, or shortness of breath at this time. Vitals were within normal limits and the temperature was 100°F on presentation. We performed routine labs, blood cultures, urinalaysis from the ileal conduit, and urine cultures (Table 1).

**Table 1:** Patient's lab results

LAB TEST	PATIENT’S VALUES	NORMAL
White blood cell count	11.18 k/uL	4-10.9 k/uL
Hemoglobin	11.0 g/dL	13.4-16.9 g/dL
Platelet count	193 k/uL	143-382 k/uL
Sodium	139 mmol/L	135-145 mmol/L
Potassium	4.9 mmol/L	3.5-5.0 mmol/L
Chloride	101 mmol/L	96-107 mmol/L
Bicarbonate	25 mmol/L	22-30 mmol/L
Urine Nitrite	Positive	Negative
Urine Leucocyte Esterase	Moderate	Negative
Urine White Blood Cell	21-50 / hpf	0-2 /hpf
Urine Red Blood Cell	3-5 /hpf	0-2 /hpf
Urine Bacteria	1+	None

**Figure 1:**
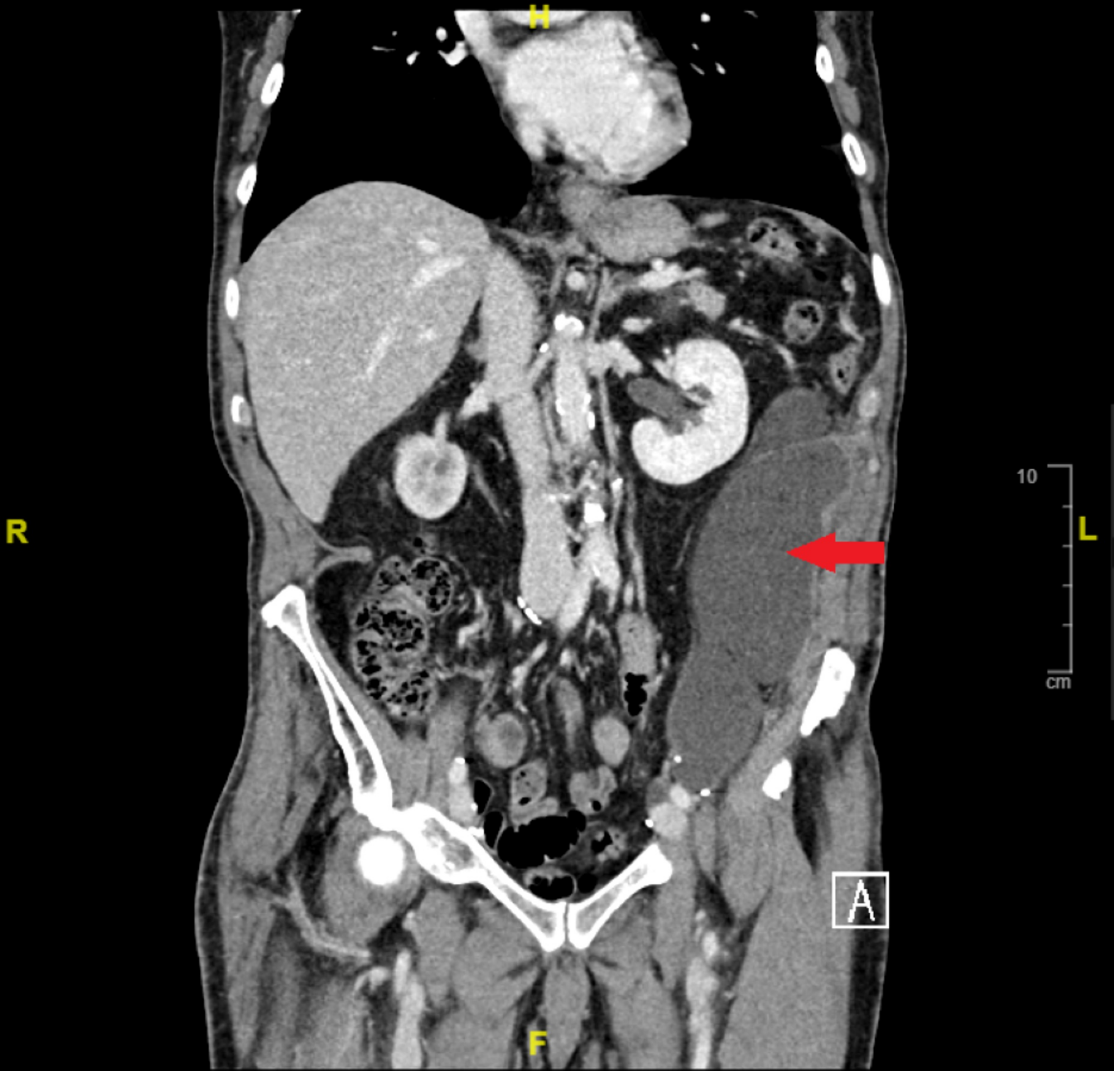
Computed tomography of the abdomen and pelvis Red arrow: Large left flank lymphocele measuring 20.4 x 9.8 x 6.2 cm with no associated gas formation or substantial surrounding inflammation.

A computed tomography of the abdomen and pelvis with contrast showed the presence of a large lymphocele (Figure 1).

We empirically started him on ceftriaxone. The patient also underwent a drain placement and fluid cultures from the lymphocele were sent. Two days later, blood cultures and fluid cultures showed the presence of S. lugdunensis with susceptibilities (Table 2).

**Table 2:** Staphylococcus lugdunensis susceptibilities from blood and fluid cultures

Staphylococcus lugdunensis		
	Sensitivity	Minimum inhibitory concentration
Penicillin	R	
Ciprofloxacin	S	<= 0.5
Clindamycin	S	<= 0.25
Tetracycline	S	<=1
Trimethoprim/Sulfa	S	<=10
Vancomycin	S	<= 0.5

Ceftriaxone was stopped and vancomycin was started. Patient was subsequently discharged two days later with 2-week course of intravenous vancomycin. Patient had resolution of his symptoms after two weeks and had negative blood cultures.

## Discussion

S. lugdunensis is a coagulase-negative staphylococci normally known to be a skin colonizer [3]. Over the years, it has shown to cause a wide variety of infections especially involving prosthetic joints and heart valves [4-5]. Bocher et al. noted that majority of the cases were found in skin abscesses, wound infections, and paronychias [3]. However, serious infections could occur from S. lugdunensis affecting prosthetic joints and heart valves. Patients infected by S. lugdunensis have shown to have concurrent comorbidities such as chronic immunosuppression, diabetes, or recent trauma [4-6]. Skin infections and abscess formations are the most common sources of S. lugdunensis infections [3]. S. lugdunensis has been noted to demonstrate antibiotic sensitivity similar to methicillin-sensitive Staphylococcus aureus [5]. 

When the diagnosis of S. lugdunensis occurs, there is difficulty in determining if it is a clinically significant infection or from contamination of the specimen or colonization of skin or mucous membranes [7]. Therefore repeat cultures are often recommended before S. lugdunensis infection can be diagnosed [8]. The use of matrix-assisted laser desorption/ionization time of flight mass spectrometry (MALDI-TOF MS) in laboratories has resulted in more accurate S. lugdunensis identification [9].

S. lugdunensis has been noted to be highly susceptible to penicillins, such as oxacillin, erythromycin, linezolid, and a wide variety of other antibiotics [3-4]. Bocher et al. state they noted no difference in antimicrobial susceptibility between isolates from hospitalized patients and isolates from general practice. S. lugdunensis produces a biofilm that makes treatment challenging even with susceptible antibiotics [3]. Such is the case with our patient, where it was resistant to penicillin. We, therefore, utilized vancomycin to be more effective. However, the data on S. lugdunensis is growing as more case reports are being published in regards to source and susceptibilities.

## Conclusions

S. lugdunensis is common skin commnesal that can result in significant clinically relevant infections when noted in abscess, heart valves, or prosthetic joints. It should be diagnosed with repeat cultures and MALDI-TOF MS in clinical laboratories. As we gain more knowledge of its susceptibilities and infectivity patterns, we can improve upon our treatment of S. lugdunensis.
